# Safe On-Boat Resuscitation by Lifeguards in COVID-19 Era: A Pilot Study Comparing Three Sets of Personal Protective Equipment

**DOI:** 10.1017/S1049023X2100011X

**Published:** 2021-01-27

**Authors:** Roberto Barcala-Furelos, Cristian Abelairas-Gómez, Alejandra Alonso-Calvete, Francisco Cano-Noguera, Aida Carballo-Fazanes, Santiago Martínez-Isasi, Antonio Rodríguez-Núñez

**Affiliations:** 1. REMOSS Research Group, Faculty of Education and Sports Sciences, University of Vigo, Pontevedra, Spain; 2. CLINURSID Network Group, Department of Psychiatry, Radiology and Public Health. University of Santiago de Compostela, Santiago de Compostela, Spain; 3. Faculty of Education Sciences, University of Santiago de Compostela, Santiago de Compostela, Spain; 4. Faculty of Sport, University of Murcia, Murcia, Spain; 5. Faculty of Nursing, University of Santiago de Compostela, Santiago de Compostela, Spain

**Keywords:** COVID-19, drowning, emergency treatment, resuscitation

## Abstract

**Introduction:**

On-boat resuscitation can be applied by lifeguards in an inflatable rescue boat (IRB). Due to Severe Acute Respiratory Syndrome Coronavirus-2 (SARS-COV-2) and recommendations for the use of personal protective equipment (PPE), prehospital care procedures need to be re-evaluated. The objective of this study was to determine how the use of PPE influences the amount of preparation time needed before beginning actual resuscitation and the quality of cardiopulmonary resuscitation (CPR; QCPR) on an IRB.

**Methods:**

Three CPR tests were performed by 14 lifeguards, in teams of two, wearing different PPE: (1) Basic PPE (B-PPE): gloves, a mask, and protective glasses; (2) Full PPE (F-PPE): B-PPE + a waterproof apron; and (3) Basic PPE + plastic blanket (B+PPE). On-boat resuscitation using a bag-valve-mask (BVM) and high efficiency particulate air (HEPA) filter was performed sailing at 20km/hour.

**Results:**

Using B-PPE takes less time and is significantly faster than F-PPE (B-PPE 17 [SD = 2] seconds versus F-PPE 69 [SD = 17] seconds; P = .001), and the use of B+PPE is slightly higher (B-PPE 17 [SD = 2] seconds versus B+PPE 34 [SD = 6] seconds; P = .002). The QCPR remained similar in all three scenarios (P >.05), reaching values over 79%.

**Conclusion:**

The use of PPE during on-board resuscitation is feasible and does not interfere with quality when performed by trained lifeguards. The use of a plastic blanket could be a quick and easy alternative to offer extra protection to lifeguards during CPR on an IRB.

## Introduction

Resuscitation in case of drowning is considered a particular circumstance.^1^ The two aspects that define the complexity of drowning cardiopulmonary resuscitation (CPR) are the asphyxial origin of the cardiac arrest^2^ and the challenging environment that often delays the onset of CPR.^3^ In this context, “time is brain,” and to quickly combat hypoxia, several studies on lifeboats have analyzed how on-boat resuscitation is feasible.

Prior to the appearance of Severe Acute Respiratory Syndrome Coronavirus-2 (SARS-COV-2) which causes coronavirus disease 2019 (COVID-19),^4^ certain studies had evaluated CPR and the use of automatic external defibrillators on inflatable rescue boats (IRB).^5-8^ However, after the appearance of COVID-19, the CPR recommendations have been updated, proposing the use of the bag-valve-mask (BVM) with a high efficiency particulate air (HEPA) filter, handled by two rescuers.^9,10^ In addition, as an anti-contagious measure, the use of personal protective equipment (PPE) has been emphasized, including at least: polycarbonate safety glasses, gloves, and a mask filtering facepiece (FFP)/N95,^10^ and for safer clinical practice, a short-sleeved apron for droplet precaution and/or a long-sleeved gown for airborne-precaution.^11^

Alternatively, either due to the absence of PPE, the low economic cost, or the peculiarities of the clinical intervention, the use of plastic blankets has been experimented with as an extra or alternative protection in the intra-hospital setting,^12-15^ and in prehospital settings, as shown by a recent pilot study with a lifeguard resuscitation simulation on the beach.^16^ In this new scenario, some resuscitation procedures are currently not recommended (eg, in-water resuscitation)^10,11^ and others are not yet certain to be applied (eg, on-boat resuscitation). For this reason and because of the health emergency, scientific societies linked to the prevention and treatment of drowning are calling for progress in research^10^ in order to try to avoid deaths that are collateral to COVID-19.

The initial hypothesis was that on-boat resuscitation on an IRB using PPE is possible and its applicability will be conditioned by two new variables: the level of PPE used and the number of rescuers on board the boat. Moreover, the use of PPE appears to take so much time to dress in special circumstances as aquatic environments.

The main objective of this pilot study has been to test how different types of PPE influence the actual starting time of CPR and its quality. In addition, the fatigue perceived by the rescuers in these new conditions and their ability to use the PPE properly have also been analyzed.

## Methods

### Study Design

**Figure 1.**
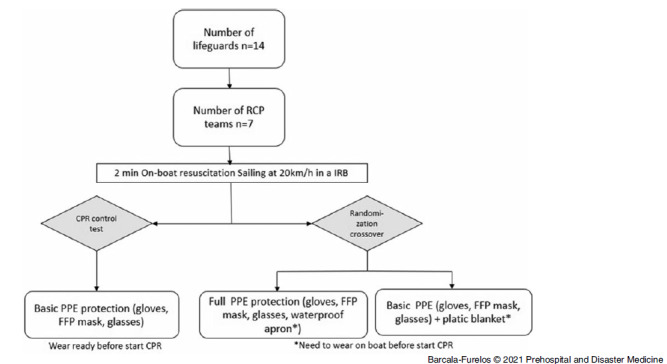
Flow Chart Design.

Abbreviations: CPR, cardiopulmonary resuscitation; PPE, personal protective equipment; FFP, filtering facepiece; IRB, inflatable rescue boat.

A comparison study of three PPE methods, using a cross-over design, was carried out to test the time difference in actually beginning on-boat resuscitation and CPR with three different levels of PPE protection (Figure 1).

### Sample

Fourteen lifeguard volunteers participated in this study. The inclusion criteria were professional lifeguards, updated according to the recommendations of the European Resuscitation Council (Niel, Belgium) Guidelines of Resuscitation (ERC-GR2015)^17^ and European Resuscitation Council COVID recommendations guidelines (ERC-COVID),^9^ who should not present any physical or psychological contraindication to carrying out the study and should authorize their participation by means of written consent. The final sample was 14 rescuers (ten men, four women). The general characteristics were: age 32 (SD = 9) years; weight 72 (SD = 14) kg; and height 173 (SD = 10) cm. This project was approved by the ethics committee of the Faculty of Education and Sport Sciences, University of Vigo, Spain (nº 03-0920).

### Roller Refresher

**Figure 2.**
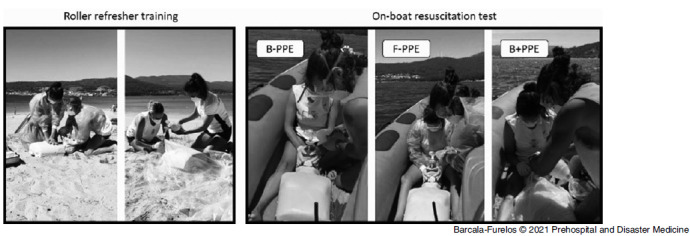
Phases of Study: On-Shore Roller Refresher Training and On-Boat Resuscitation Test.

Abbreviations: PPE, personal protective equipment; B-PPE, basic PPE (gloves, glasses, and FFP mask); F-PPE, full PPE (gloves, glasses, FFP mask, and waterproof coat); B+PPE, basic PPE + plastic blanket.

A refresher was carried out before the intervention in order to standardize skills and become familiar with the PPE equipment during CPR with BVM (+HEPA filter). This roller refresher lasted 40 minutes and was organized as follows: Part 1 - Explanation and training in the dressing and use of PPE (20 minutes); Part 2 - CPR training with complete PPE in a team of two rescuers (10 minutes); and Part 3 - CPR training with basic PPE (B-PPE) and plastic blanket (B+PPE) in a team of two rescuers (10 minutes). This training was conducted by a nurse instructor accredited by the Spanish Resuscitation Council (Madrid, Spain; Figure 2).

### Controlled On-Board Resuscitation (Supplementary Video Online)

Three CPR tests were performed, following the technical recommendations for the ERC-COVID^9^ resuscitation, using a sequence in accordance with ERC-GR2015 drowning recommendations.^1^ The sequence consisted of five rescue ventilations (V), followed by cycles of 30 chest compressions (CC) and two Vs, with a duration of two minutes, and the following were compared:

- Test with Basic PPE (B-PPE): Nitrile gloves, FFP mask, and protective glasses;
- Test with Full PPE (F-PPE): Nitrile gloves, FFP mask, protective glasses, and waterproof gown; and
- Test with Basic PPE + plastic blanket (B+PPE): Nitrile gloves, FFP mask, protective glasses, and transparent plastic blanket, approximately 250cm long by 150cm wide, according to a previous pilot study.^16^

In each CPR, an Ambu Mark IV adult BVM (Ambu; Ballerup, Denmark) with an Ambu HEPA filter (Ambu; Ballerup, Denmark) was used on a Laerdal Little Anne QCPR manikin (Laerdal; Stavanger, Norway).

The tests were performed on Broña Beach (Serra de Outes, A Coruña - Spain), GPS positioning: Latitude 42.801747, Longitude -8.929523. In order to be more realistic, each test began having stopped the boat and accelerating at a cruising speed of 20km/hour which was maintained until the end of the test. The IRB model was a Valiant DR-450 (Vila Nova de Cerveira, Portugal), 4.5 meters long and 1.94 meters wide. The weather conditions included a calm sea (0-2 Douglas scale), a light wind between 12 and 19 km/hour (3 Beaufort scale), at an ambient temperature of 22ºC. The weather data were reported by the local weather agency (Meteogalicia; Santiago de Compostela, Spain).

### Variables

Four groups of variables were analyzed: (1) time to start resuscitation; (2) quality of resuscitation; (3) perceived fatigue during resuscitation; and (4) skill in the use of PPE.

#### Time to Beginning of CPR

The time in seconds (s) was counted from the moment the victim was indicated as being in cardio-respiratory arrest to the start of the first rescue ventilation.

#### Cardiopulmonary Resuscitation

Three resuscitation variables were analyzed: (1) the quality of the CC in %; (2) the effective V (EV) in %: EV was understood as being when the victim’s chest is clearly raised and it provides a positive record in the analysis software; and (3) quality of the CPR (Q-CPR): this is the overall result of CPR estimated by the Laerdal APP CPR instructor software (Laerdal; Stavanger, Norway) installed on an iPhone 7 (Apple Inc.; Cupertino, California USA), connected by Bluetooth to the Little Anne QCPR manikin, programmed according to ERC-GL2015.

#### Rating of Perceived Exertion

The rating of perceived exertion (RPE) was recorded (measurement of the range 0/10 - sub/maximal).^18^ Previously, the lifeguards were trained in the understanding and use of this scale.

#### Skill in the Use of Waterproof Protection

**Figure 3.**
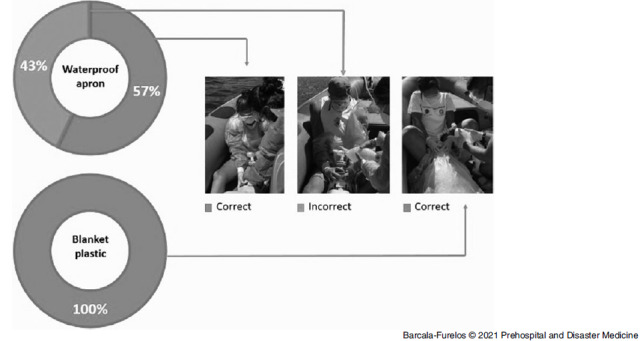
Dexterity in the Use of Waterproof Apron and Plastic Blanket.

The skill/correction in dressing the waterproof apron in the F-PPE of each lifeguard and the skill in placing the plastic blanket during the B+PPE by each team of lifeguards was subjectively evaluated. The dichotomous variable considered to be “correct dexterity” is when it provided a complete waterproof barrier between the victim and the lifeguard. If this did not occur, it was considered as “incorrect dexterity” (Figure 3).

### Statistical Analysis

All statistical analyses were performed with SPSS for Windows, version 22 (IBM Corp.; Armonk, New York USA). The Shapiro-Wilk test was used to evaluate the normality of the data. The repeated measures ANOVA test with Bonferroni correction was used to compare the parametric variables and the Friedman test was used for non-parametric variables. A significance of P <.05 was established for all analyses on parametric variables and P <.017 on non-parametric variables.

## Results

### Time Variables

In the analysis of time to initiation of CPR, it was found that rescuers previously equipped with B-PPE took 17 seconds to initiate CPR. This result was an improvement of 52 seconds compared to F-PEE (B-PPE 17 [SD = 2] seconds versus F-PPE 69 [SD = 17] seconds; P = .001) and was 17 seconds faster than B+PPE (B-PPE 17 [SD = 2] seconds versus B+PEE 34 [SD = 6] seconds; P = .002). On analyzing PPE with full waterproof protection (B+PPE or F-PPE), wearing a plastic blanket saved more than half-a-minute compared to wearing a waterproof apron on-boat (B+PPE 34 [SD = 6] seconds versus F-PPE 69 [SD = 17] seconds; P = .006; Table 1).

**Table 1.** Results of the Time, CPR, and RPE Variables

Variables	Basic-PPE		Full-PPE		Basic-PPE + PB			
	Mean (SD)	CI (95%)	Mean (SD)	CI (95%)	Mean (SD)	CI (95%)		
**Time to Start CPR (s)**	**17** (SD = 2)	15-19	**69** (SD = 16)	55-84	**34** (SD = 6)	28-40	P <.001^a^	B-PPE vs F-PPE: P = .001
								B-PPE vs B+PPE: P = .002
								F-PPE vs B+PPE: P = .006
**Global QCPR (%)**	**86** (SD = 16)	72-101	**94** (SD = 3)	92-97	**91** (SD = 5)	87-96	P = .31^b^	B-PPE vs F-PPE: P = .17
								B-PPE vs B+PPE: P = .60
								F-PPE vs B+PPE: P = .07
**QCC (%)**	**86** (SD = 22)	66-106	**99** (SD = 1)	98-100	**97** (SD = 3)	95-100	P = .12^b^	B-PPE vs F-PPE: P = .10
								B-PPE vs B+PPE: P = .07
								F-PPE vs B+PPE: P = .14
**EV (%)**	**90** (SD = 7)	84-96	**83** (SD = 12)	71-94	**79** (SD = 15)	65-93	P = .20^a^	B-PPE vs F-PPE: P = .38
								B-PPE vs B+PPE: P = .39
								F-PPE vs B+PPE: P = 1.00
**RPE (CC)**	**3** (SD = 1)	3-4	**3** (SD = 1)	2-4	**3** (SD = 1)	2-4	P = .54^a^	B-PPE vs F-PPE: P = 1.00
								B-PPE vs B+PPE: P = 1.00
								F-PPE vs B+PPE: P = 1.00
**RPE (V)**	**2** (SD = 1)	2-3	**3** (SD = 1)	2-3	**2** (SD = 1)	2-3	P = .61^b^	B-PPE vs F-PPE: P = .32
								B-PPE vs B+PPE: P = .32
								F-PPE vs B+PPE: P = .66

Abbreviations: CPR, cardiopulmonary resuscitation; QCPR, quality of cardiopulmonary resuscitation in percentage; QCC, quality of chest compressions in percentage; EV, effective ventilations in percentage; RPE, rating of perceive exertion (0-10 scale); CC, chest compression; V, ventilation; B-PPE, basic PPE (gloves, glasses, and FFP mask); F-PPE, full PPE (gloves, glasses, FFP mask, and waterproof coat); B+PPE, basic PPE + plastic blanket; PB, plastic blanket; FFP, filtering facepiece.

a ANOVA of repeated measures with Bonferroni correction.

b Friedman’s repeated measures with Bonferroni correction.

### CPR Variables

**Figure 4.**
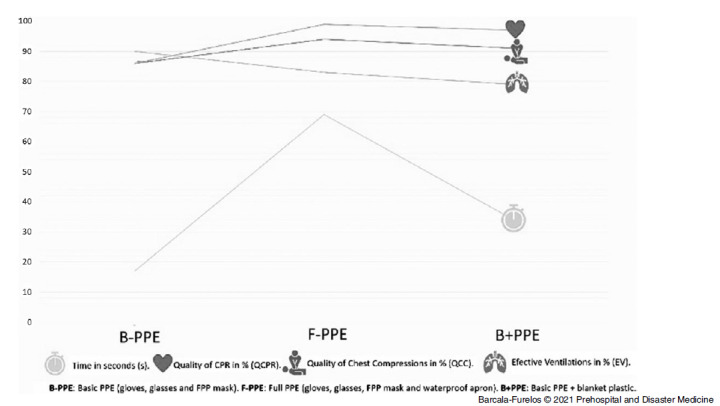
Visual Chart of Variables: Time and CPR.

Abbreviations: PPE, personal protective equipment; B-PPE, basic PPE (gloves, glasses, and FFP mask); F-PPE, full PPE (gloves, glasses, FFP mask, and waterproof coat); B+PPE, basic PPE + plastic blanket; QCPR, quality of cardiopulmonary resuscitation; QCC, quality of chest compressions; EV, effective ventilations.

The CPR was of equally good quality in all three scenarios (B-PPE versus F-PPE versus B+PPE; P >.05). The rescuers obtained values above 79% in all the variables analyzed. There was a non-significant trend (P >.05) of a seven percent decrease in the percentage of EV when using F-PPE and an 11% decrease with B+PPE, compared to B-PPE, which obtained the highest value (90%; Table 1; Figure 4).

### RPE Variable

The RPE was similar in the conditions of all three scenarios (P >.05), with low scores as the values ranged from two to three on a maximum scale of ten (Table 1).

### Skill in the Use of Waterproof Protection Variable

All teams (100%) were able to use the plastic blanket correctly and to keep it stable throughout the test. However, 43% were not able to correctly don the waterproof apron.

## Discussion

Rescuer protection is essential in any emergency. In addition to the usual risks (usually traumatic), now there is the risk of COVID-19 infection. For this reason, it is suggested that professionals should use isolation devices which prevent contact and virus inhalation if the victim is infected. So far, recommendations for resuscitation have been based on the most common “medical” settings (ie, hospital or ambulance), without yet having evaluated the options for PPE in other less common and less controlled environments (such as lifesaving situations) in which CPR is also performed. Therefore, this study had the novel objective of evaluating three levels of protection during the resuscitation by rescuers on an IRB. In the controlled simulation conditions in a real environment in which the tests were carried out, it was observed that: (1) there was less loss of time at the start of on-boat resuscitation using the BVM and HEPA filter, with a basic level of protection; (2) the use of an extra waterproof barrier such as a plastic blanket seemed faster and easier than a conventional waterproof apron; (3) CPR quality was not affected by the level of protection used; and (4) finally, on-boat CPR with two rescuers did not generate much fatigue amongst trained lifeguards.

Drowning is considered a public health issue by the World Health Organization (WHO; Geneva, Switzerland),^19^ and lifeguards are recognized as the first barrier to prevention and intervention. The IRB is commonly used in lifeguarding as it is small, safe, fast, and easy to use, and is common in surveillance and rescue near the coast.^5^ The use of IRBs in the event of drowning can gain valuable time in an incident in which every second counts. The systematic review and meta-analysis by Quan, et al^20^ found that immersion time is the most influential factor in the prognosis of the victim. Stopping the drowning process quickly^2^ and initiating on-boat resuscitation would avoid the time delay involved in the rescue and transfer to land without life support. Another finding by Quan, et al^20^ is the favorable outcomes witnessed thanks to the shorter Emergency Medical Service response times. This evidence reinforces the importance of on-boat resuscitation, which is not a common practice but is definitely possible,^7,21^ and therefore needs planning and training.

At present, any unknown victim of cardio-respiratory arrest will be considered a potential carrier of SARS-COV-2, and in the case of rescuers, exposure to the risk of contagion may be high since beaches are a place with a large concentration of bathers and rescue techniques inevitably require direct contact.^11^ The risk may be reduced depending on the PPE that can be used.^11^ From a theoretical perspective, the most complete option of F-PPE could be used on the IRBs; however, it would not be realistic for two reasons: (1) it is neither viable nor safe to patrol with a waterproof gown while awaiting an incident requiring CPR, since it is a low-probability event (it represents just 0.02% of actions carried out by lifeguards);^22^ and (2) the time spent wearing the waterproof gown on a boat, as well as the probability of doing it incorrectly, is a sufficiently important limitation affecting this choice of PPE.

Maintaining on-boat resuscitation as a protocol on IRBs involves starting CPR with a basic protection (B-PPE) and ventilating using HEPA-filtered BVMs. However, this procedure does not offer the greatest protection against possible infection and should be assessed by rescue agencies, and the rescuers themselves, to weigh up the risk they are taking considering epidemiological data on local incidence of the virus, the age of the rescuer (usually young) or his/her previous health status (eg, previous pathology, possible immunity due to having previously overcome COVID-19, or other factors), or the type of victim (eg, a child) and the rescue conditions (eg, a short time underwater). An intermediate position could be the use of B-PPE in combination with a plastic blanket (B+PPE) so as to create an insulating barrier between the rescuers and the victim. According to these results, this extra protection allows quick positioning on the patient and does not affect the QCPR.

A fundamental criterion for the decision to continue with on-boat resuscitation is to know whether it is possible to perform quality CPR with the PPE. Studies prior to the pandemic have shown good performance by lifeguards or fishermen during on-boat resuscitation, although the QCPR was conditioned by the size of the boat,^6^ the waves,^6^ the wind, or the speed,^5,23^ but until now, neither the PPE variable nor on-boat resuscitation in teams (by two lifeguards) had been introduced. These findings show results in V and CC above 70%, a value arbitrarily assumed in numerous studies as the cut-off point in CPR quality.^24^ Similar results were found in a Spanish study on an IRB,^5^ with the same maritime conditions and at a very similar speed (10knots/18.52Km/hour) in comparison with the current study (11knots/20Km/hour). The main difference between both studies was the RPE. In the case of a lone rescuer, the perceived effort was five (Heavy/Strong big-major effort) on the RPE scale,^18^ and when there were two rescuers (ie, in this research), the RPE did not exceed two (a light effort) for the rescuer performing V and three (moderate effort) for the CC rescuer. It seems, therefore, that CPR by two lifeguards has a number of advantages, at least in terms of fatigue, although the crew of an IRB is usually made up of two people (skipper and lifeguard) since a conditioning factor is the limited space.^7^ This circumstance could be a limitation for team resuscitation on small IRB models.

Correct PPE donning and doffing is not easy and requires specific training,^11^ and misuse may lead to a false sense of security.^25^ Wind and waves are common circumstances at sea, and tests performed with mild gusts of wind prevented several rescuers from wearing the waterproof gown properly.

This study has a practical and direct impact on lifeguards, regarding how to deal with the most critical situation of drowning (ie, cardio-respiratory arrest). Europe is immersed in the first summer of the COVID-19 Era and this has not prevented the beaches of Mediterranean countries from continuing to be a meeting point for bathers and the scene of potential drownings. Summer will be at the end of 2020 in the Southern Hemisphere, so lifeguards need evidence-based guidance to intervene as safely as possible, in a context in which there are no previous experience and therefore recommendations must be adapted to each specific environment. Preliminary results are offered here in three aspects relevant to on-boat resuscitation: (1) whether it is feasible with current recommendations; (2) how precious time can be saved so as not to delay assistance; and (3) how to protect rescuers to prevent contagion.

## Study Limitations

This work presents limitations that should be pointed out. Firstly, it is a pilot study carried out in controlled simulation conditions in favorable weather conditions, with a small, local sample of lifeguards. The same tests with other maritime conditions, human or material resources, could obtain different results. The major limitation is the use of a dummy. In a real victim, the difficulty of resuscitation will be different and more complex.

Three levels of PPE were investigated, based on current knowledge and recommendations for the prevention of COVID-19 during CPR. However, the actual risk is not possible to fully measure with this study.

To the authors’ knowledge, this is the first research work that aims to assess the feasibility of on-boat resuscitation during the COVID-19 Era, so there may be other limitations not described and not yet known by the authors.

## Conclusions

The use of PPE during on-board CPR is feasible and does not interfere with quality when performed by trained lifeguards. The use of B-PPE allows for rapid initiation of CPR. The use of PPE which requires wearing a waterproof apron on board is a significant loss of time that delays the start of CPR. The use of a plastic blanket could be a quick and easy alternative to offer extra protection to the lifeguards during on-boat resuscitation on an IRB.

This pilot study could help Lifesavers’ Organizations to define their rescue and resuscitation protocols, based on the local situation, pandemic level, experience, training, and available materials.

## Supplementary material

For supplementary material accompanying this paper visit https://doi.org/10.1017/S1049023X2100011X. click here to view supplementary material

## References

[1] Truhlář A , Deakin CD , Soar J , et al. European Resuscitation Council Guidelines for Resuscitation 2015: Section 4. Cardiac arrest in special circumstances. Resuscitation. 2015;95:148–201. 26477412 10.1016/j.resuscitation.2015.07.017

[2] Szpilman D , Bierens JJLM , Handley AJ , Orlowski JP . Drowning. N Engl J Med. 2012;366(22):2102–2110. 22646632 10.1056/NEJMra1013317

[3] Winkler BE , Eff AM , Ehrmann U , et al. Effectiveness and safety of in-water resuscitation performed by lifeguards and laypersons: a crossover manikin study. Prehosp Emerg Care. 2013;17(3):409–415. 23734993 10.3109/10903127.2013.792892

[4] World Health Organization. Naming the coronavirus disease (COVID-2019) and the virus that causes it. https://www.who.int/emergencies/diseases/novel-coronavirus-2019/technical-guidance/naming-the-coronavirus-disease-(covid-2019)-and-the-virus-that-causes-it. Accessed May 11, 2020.

[5] Barcala-Furelos R , Abelairas-Gomez C , Palacios-Aguilar J , et al. Can surf-lifeguards perform a quality cardiopulmonary resuscitation sailing on a lifeboat? A quasi-experimental study. Emerg Med J. 2017;34(6):370–375. 28130348 10.1136/emermed-2016-205952

[6] Tipton M , David G , Eglin C , Golden F. Basic life support on small boats at sea. Resuscitation. 2007;75(2):332–337. 17574722 10.1016/j.resuscitation.2007.04.027

[7] Seesink J , Nieuwenburg SAV , van der Linden T , Bierens JJLM . Circumstances, outcome and quality of cardiopulmonary resuscitation by lifeboat crews. Resuscitation. 2019;142:104–110. 31351088 10.1016/j.resuscitation.2019.07.012

[8] de Vries W , Bierens JJLM , Maas MWM. Moderate sea states do not influence the application of an AED in rigid inflatable boats. Resuscitation. 2006;70(2):247–253. 16806638 10.1016/j.resuscitation.2006.01.008

[9] Nolan JP , Monsieurs KG , Bossaert L , et al. European Resuscitation Council COVID-19 guidelines executive summary. Resuscitation. 2020;153:45–55. 32525022 10.1016/j.resuscitation.2020.06.001 PMC7276132

[10] Queiroga C , Bierens J , Dunne C , Manino L , van der Linden T , Mecrow T. Position statement on behalf of the International Drowning Researchers’ Alliance [IDRA], International Life Saving Federation – Medical Committee [ILS-MC] and International Maritime Rescue Federation [IMRF]. Resuscitation of the drowned person in the era of COVID-19 disease: a common ground for recommendations, identification of research needs and a global call to action. http://idra.world/portfolio/covid_cpr_guidelines/. Accessed October 2020.

[11] Barcala-Furelos R , Aranda-García S , Abelairas-Gómez C , et al. Occupational health recommendations for lifeguards in aquatic emergencies in the Covid-19 era: prevention, rescue and resuscitation. Rev Esp Salud Publica. 2020;94. PMC11583182 32601267

[12] Matava CT , Yu J , Denning S. Clear plastic drapes may be effective at limiting aerosolization and droplet spray during extubation: implications for COVID-19. Can J Anaesth J Can Anesth. 2020;67(7):902–904. 10.1007/s12630-020-01649-w PMC7124129 32246431

[13] Chow VLY , Chan JYW , Ho VWY , et al. Tracheostomy during COVID-19 pandemic—novel approach. Head Neck. https://www.ncbi.nlm.nih.gov/pmc/articles/PMC7267533/. Accessed June 28, 2020. 10.1002/hed.26234 PMC7267533 32358855

[14] Brown S , Patrao F , Verma S , Lean A , Flack S , Polaner D. Barrier system for airway management of COVID-19 patients. Anesth Analg. 2020;131(1):e34–e35. 33035017 10.1213/ANE.0000000000004876 PMC7179052

[15] Allen B , Gardner C , O’Neill C , Gibbs M. Use of drape/patient covering during potentially aerosolizing procedures. Am J Emerg Med. 2021;39:227–228. 32402501 10.1016/j.ajem.2020.05.007 PMC7204666

[16] Barcala-Furelos R , Szpilman D , Abelairas-Gómez C , et al. Plastic blanket drowning kit: a protection barrier to immediate resuscitation at the beach in the Covid-19 era. A pilot study. Am J Emerg Med. 2020;38(11):2395–2399. 33039225 10.1016/j.ajem.2020.08.101 PMC7492152

[17] Monsieurs KG , Nolan JP , Bossaert LL , et al. European Resuscitation Council Guidelines for Resuscitation 2015: Section 1. Executive summary. Resuscitation. 2015;95:1–80. 26477410 10.1016/j.resuscitation.2015.07.038

[18] Foster C , Florhaug JA , Franklin J , et al. A new approach to monitoring exercise training. J Strength Cond Res. 2001;15(1):109–115. 11708692

[19] World Health Organization. Preventing Drowning: An Implementation Guide. Geneva, Switzerland: World Health Organization; 2017:105.

[20] Quan L , Bierens JJLM , Lis R , Rowhani-Rahbar A , Morley P , Perkins GD. Predicting outcome of drowning at the scene: a systematic review and meta-analyses. Resuscitation. 2016;104:63–75. 27154004 10.1016/j.resuscitation.2016.04.006

[21] Kingdon D , Stapleton E , Stahl E. Successful resuscitation: novel partnership between paramedics and US Coast Guard. Prehosp Emerg Care. 2016;20(3):432–438. 26808462 10.3109/10903127.2015.1111478

[22] Szpilman D , de Barros Oliveira R , Mocellin O , Webber J. Is drowning a mere matter of resuscitation? Resuscitation. 2018;129:103–106. 29928958 10.1016/j.resuscitation.2018.06.018

[23] Fernández Méndez F , Barcala-Furelos R , Fungueiriño-Suárez R , Mecías-Calvo M , Abelairas-Gómez C , Rodríguez-Núñez A. Cardiopulmonary resuscitation quality during navigation in inshore fishing boats: a pilot study with fishermen. Am J Emerg Med. 2015;33(11):1705–1707. 10.1016/j.ajem.2015.08.018 26349775

[24] Perkins GD , Colquhoun M , Simons R. ABC of Resuscitation. 5th ed London UK: BMJ Books; 2004:97–101.

[25] Amoroso D , Poncetti GL , Regueira ES , Pocebon LZ , Guimarães HP. Recomendações para Reutilização Cíclica Racional de Equipamentos de Proteção Individual Durante a Pandemia por COVID-19. 2020:8.

